# Antimicrobial Resistance Profiles of Human Commensal *Neisseria* Species

**DOI:** 10.3390/antibiotics10050538

**Published:** 2021-05-06

**Authors:** Maira Goytia, Symone T. Thompson, Skylar V. L. Jordan, Kacey A. King

**Affiliations:** Department of Biology, Spelman College, Atlanta, GA 30314, USA; st952@cornell.edu (S.T.T.); sjorda29@spelman.edu (S.V.L.J.); kking80@student.gsu.edu (K.A.K.)

**Keywords:** Commensal bacteria, *Neisseria*, antimicrobial resistance, multidrug resistance

## Abstract

Pathogenic *Neisseria gonorrhoeae* causes the sexually transmitted infection gonorrhea. *N. gonorrhoeae* has evolved high levels of antimicrobial resistance (AR) leading to therapeutic failures even in dual-therapy treatment with azithromycin and ceftriaxone. AR mechanisms can be acquired by genetic transfer from closely related species, such as naturally competent commensal *Neisseria* species. At present, little is known about the antimicrobial resistance profiles of commensal *Neisseria*. Here, we characterized the phenotypic resistance profile of four commensal *Neisseria* species (*N. lactamica*, *N. cinerea*, *N. mucosa*, and *N. elongata*) against 10 commonly used antibiotics, and compared their profiles to 4 *N. gonorrhoeae* strains, using disk diffusion and minimal inhibitory concentration assays. Overall, we observed that 3 of the 4 commensals were more resistant to several antibiotics than pathogenic *N. gonorrhoeae* strains. Next, we compared publicly available protein sequences of known AR genes, including penicillin-binding-protein 2 (PBP2) from commensals and *N. gonorrhoeae* strains. We found mutations in PBP2 known to confer resistance in *N. gonorrhoeae* also present in commensal *Neisseria* sequences. Our results suggest that commensal *Neisseria* have unexplored antibiotic resistance gene pools that may be exchanged with pathogenic *N. gonorrhoeae*, possibly impairing drug development and clinical treatment.

## 1. Introduction

*Neisseria gonorrhoeae*, the etiologic agent of gonorrhea, is the second most commonly reported bacterial sexually transmitted infection in the US [1], and a worldwide public health concern. The World Health Organization (WHO) estimates there were 87 million new cases globally of *N. gonorrhoeae* in 2016 [2]; this incidence is increasing in many countries including the USA [3]. The Centers for Disease Control and Prevention (CDC) classifies *N. gonorrhoeae* as an “urgent threat” due to the emergence of antimicrobial resistance (AR) and multidrug resistance (MDR) [4,5,6,7,8,9]. The spread of AR has led to increasing rates of untreatable gonorrhea [10], including reports of untreatable and harder to treat pharyngeal *N. gonorrhoeae*, acquired through oral sex [11,12,13], where *N. gonorrhoeae* shares the environment with closely related commensal *Neisseria*.

Pathogenic and commensal *Neisseria* species exchange and transfer genes via natural competence and transformation [14]. *Neisseria* will exchange and transfer genes at high rates, as long as they share an identical or similar DNA uptake Sequences (DUS) and the corresponding DNA import complex [15,16,17,18]. Hence, in this study, we characterize the antimicrobial resistance profiles of commensal *Neisseria* species and explore their potential role as antibiotic resistance gene reservoirs for pathogenic *Neisseria* species. We selected and characterized commensal *Neisseria* species across a spectrum of genetic relatedness, including *N. lactamica*, *N. elongata*, *N. cinerea*, and *N. mucosa*. Of these, *N. lactamica* is the closest relative to the pathogens *N. gonorrhoeae* and *N. meningitidis*, while *N. elongata* appears to be the most distant relative to the pathogens [19,20,21]. *N. cinerea* is more closely related to *N. lactamica*; *N. mucosa* is closely related to the common ancestor of *N. lactamica* and *N. cinerea*. We also used the *N. gonorrhoeae* FA19 strain [22], susceptible to antibiotics, as our reference strain, while strains MS11, H041, and F89 are representatives of pathogenic resistant strains for several antibiotics. We tested commensal and pathogenic strains against a panel of 10 antibiotics that are commonly or were previously used, or likely to be used, in a therapeutic context. We used disk diffusion (DDA) and minimal inhibitory concentration (MIC) assays, and show that commensal *Neisseria* display increased antimicrobial resistance profiles to widely used antibiotics, including the first line dual-therapy drugs, azithromycin and ceftriaxone, compared to pathogenic *N. gonorrhoeae* strains. Notably, when analyzing AR-associated genes, including *penA* sequences, which encode the Penicillin Binding Protein 2 (PBP2), we identified several mutations in some commensal *Neisseria* species that are known to cause resistance in *N. gonorrhoeae*. We discuss these findings in light of the potential for commensal *Neisseria* to function as de facto reservoirs of antibiotic resistance genes for the pathogenic *N. gonorrhoeae*.

## 2. Results

### 2.1. Commensal Neisseria Display Increased Resistance Levels to Several Antibiotics, Detected by Disk Diffusion Assays

**Figure 1:**
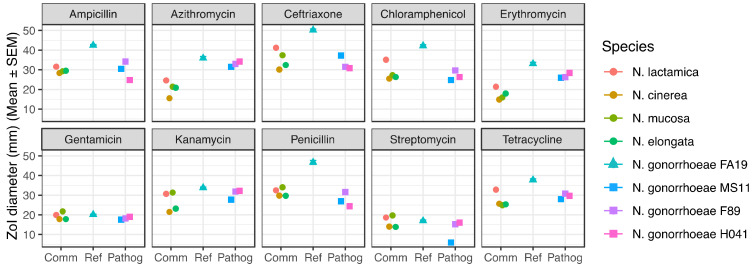
Antibiotic disk diffusion assays (DDA) on 10 antibiotics demonstrate antibiotic resistance profiles of *Neisseria* species, grouped as commensal (Comm, circles), reference (Ref, triangles) and pathogenic (Pathog, squares). Means (± SEM) of zone of inhibition (ZoI) diameters (mm) from 3 independent experiments, each performed with three biological replicates are shown. Comm, commensal; Ref, reference *N. gonorrhoeae* FA19; Pathog, pathogenic.

In order to assess the levels of antibiotic resistance of commensal *Neisseria*, we performed disk diffusion assays (DDA) with 10 commonly used antibiotics, including 3 beta-lactams and 7 protein synthesis inhibitors as described in the Methods. Our DDA results revealed that some commensal *Neisseria* species were particularly resistant to azithromycin and ceftriaxone (Figure 1 and Supplementary Table S1), the first-line treatment drugs against *N. gonorrhoeae*, as well as to erythromycin, the antibiotic applied on newborns’ eyes to prevent conjunctivitis neonatorum. All commensal species displayed increased resistance to azithromycin as evidenced by the smaller zone of inhibition (ZoI) diameters (ZoI in the range 15.5–24.6 mm) than any of the *N. gonorrhoeae* strains (ZoI in the range 31.5–35.9 mm), including the highly resistant strains *N. gonorrhoeae* F89 and H041. For ceftriaxone, only *N. lactamica* displayed susceptibility levels similar to *N. gonorrhoeae* FA19 strain, with ZoI of 41.2 mm and 50.2 mm, respectively. Commensals *N. cinerea* and *N. elongata* were as resistant to ceftriaxone (ZoI 30.1 mm and 32.4 mm, respectively) as the resistant *N. gonorrhoeae* F89 and H041 (ZoI 31.5 mm and 30.8 mm, respectively), identified as models of therapeutic failure for ceftriaxone treatments. According to the CLSI guidelines, ZoI > 35 mm suggest that *N. gonorrhoeae* were susceptible to ceftriaxone [23]; hence, only *N. lactamica*, *N. mucosa* and *N. gonorrhoeae* FA19 can be considered susceptible to ceftriaxone; all other strains tested displayed resistance to it. The resistance of commensals to erythromycin resembled azithromycin’s pattern where all commensals displayed increased erythromycin resistance levels (ZoI in the range 14.9–21.4 mm) compared to *N. gonorrhoeae* FA19 or the other *N. gonorrhoeae* strains tested (ZoI in the range 26.0–33.1 mm). Commensal *Neisseria* displayed a wide dispersion in erythromycin resistance levels.

Commensal *Neisseria* species displayed increased resistance to penicillin and ampicillin (ZoI in the range 29.7–34.0 mm and 28.3–31.6 mm, respectively) compared to the susceptible *N. gonorrhoeae* FA19 strain (ZoI 46.7 mm and 42.5 mm, respectively). According to the interpretive standards published by the CLSI [23], *N. gonorrhoeae* were considered resistant to penicillin when ZoI < 26 mm, such as *N. gonorrhoeae* MS11 and H041.

**Figure 2:**
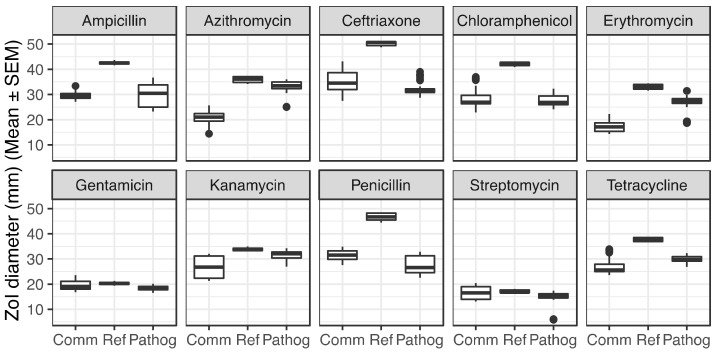
Zones of inhibition diameters (in mm) per group of species, comparing all commensals (Comm) to the reference strain *N. gonorrhoeae* FA19 (Ref), and to the group of resistant and pathogenic *N. gonorrhoeae* strains MS11, F89, and H041 (Pathog).

Similarly, *N. cinerea*, *N. mucosa*, and *N. elongata*, but not *N. lactamica*, displayed higher resistance to chloramphenicol (ZoI in the range 25.6–27.3 mm) than the susceptible *N. gonorrhoeae* FA19 (ZoI of 42.2 mm). The other *N. gonorrhoeae* strains (MS11, F89, H041) displayed ZoI to chloramphenicol similar to the commensal species. *N. cinerea*, *N. mucosa*, and *N. elongata*, but not *N. lactamica*, were more resistant to tetracycline (ZoI in the range 24.9–25.6 mm) than *N. gonorrhoeae* FA19 (ZoI of 37.8 mm). Streptomycin was the only antimicrobial tested for which we did not observe a statistically significant (adjusted *p*-value > 0.0001, One-way ANOVA) difference between the ZoI diameters of commensals and *N. gonorrhoeae* FA19 (Figure 2, Table 1 and Table 2). Only two commensals, *N. cinerea* and *N. elongata* displayed higher kanamycin resistance levels (ZoI of 21.5 and 23.1 mm, respectively) than *N. gonorrhoeae* FA19 (ZoI of 33.8 mm). It is important to note that the ZoI diameters of *N. cinerea*, *N. mucosa*, and *N. elongata* were found to be at least 33% smaller than those of *N. gonorrhoeae* FA19, for ampicillin, ceftriaxone, azithromycin, erythromycin, and chloramphenicol, and tetracycline, reflecting a wide gap in resistance levels between these 3 commensals and the susceptible *N. gonorrhoeae* FA19 strain (Supplementary Table S1).

Interestingly, both groups of commensal and pathogenic strains displayed similar levels of resistance to gentamicin (ZoI in the range 17.8–21.8 mm and 17.5–20.2 mm, respectively), a possible alternative to treat *N. gonorrhoeae* (Figure 1 and Supplementary Table S1). These ZoI to gentamicin were slightly larger than the ZoI ≥ 16 mm categorized as the limit for susceptibility to gentamicin [24]. This suggests that the *Neisseria* species tested do not currently display antibiotic resistance against gentamicin, nor should they be considered a reservoir of gentamicin resistance. However, the small difference between the threshold of susceptibility (16 mm) and the range of ZoI observed (in the range 17.5–21.8 mm) suggests that a mutation, even if slightly decreasing the susceptibility level to gentamicin, could render these species resistant, and a gentamicin treatment ineffective.

For each antibiotic, we performed a One-way ANOVA statistical test, and observed that when comparing the commensals as a group to *N. gonorrhoeae* FA19, commensals were more resistant to 8 out of 10 antibiotics tested (except for streptomycin and gentamicin, adjusted *p*-value > 0.001). The difference was statistically significant (adjusted *p*-value < 0.0001) (Figure 2, Table 1 and Table 2). In addition, for antibiotics azithromycin and erythromycin, the difference in mean for commensals to pathogenic is a third or more than the mean ZoI diameter of the pathogenic strains, suggesting also that the commensals are more resistant than the resistant strains for azithromycin and erythromycin. It is important to note that as seen in Figure 1, there is a range of antimicrobial resistance level among commensals for azithromycin and erythromycin, with *N. lactamica* being more susceptible compared to the other commensals. Similarly, it is important to note that while *N. gonorrhoeae* MS11, F89, and H041 are labelled “resistant” strains, they are not resistant to all antibiotics.

Overall, *N. cinerea*, *N. mucosa*, and *N. elongata* appeared more resistant to antibiotics than *N. lactamica*, which is more closely related to *N. gonorrhoeae* and *N. meningitidis*. As shown in Figure 1, *N. lactamica* was the most susceptible of the four commensals to seven of the 10 antibiotics tested and the second-most susceptible in the three remaining antibiotics (see Figure 1). Therefore, *N. lactamica* was most similar in its antibiotic susceptibility profile to the susceptible pathogenic *Neisseria gonorrhoeae* FA19. This suggests that the other commensal strains—*N. cinerea*, *N. mucosa*, *and N. elongata*—may have distinct antimicrobial resistance mechanisms than *N. gonorrhoeae* FA19. Indeed, the profile of resistance for these 3 commensal species was very different from *N. lactamica* and the 4 *N. gonorrhoeae* strains, possibly suggesting that these commensals carry mutations and/or genes that confer increased antimicrobial resistance, in ways that have not been observed in *N. gonorrhoeae*. This requires further exploration of genomic data from commensal *Neisseria* species, both in known antimicrobial resistance genes and in regions that are not known to confer antimicrobial resistance.

### 2.2. Commensal Neisseria Display Increased Resistance Levels to Several Antibiotics, Detected by Minimal Inhibitory Concentrations

**Figure 3:**
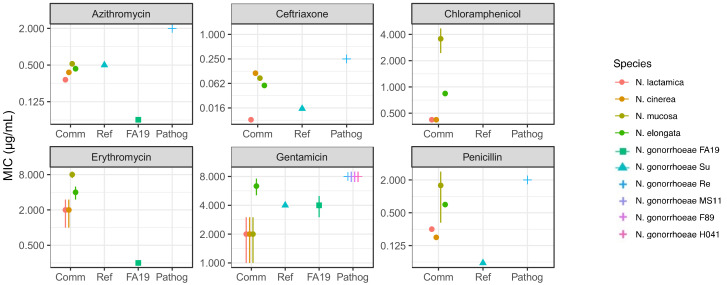
Minimal Inhibitory Concentrations (µg/mL) of several antibiotics in commensal (Comm, circles) *Neisseria* species compared to *N. gonorrhoeae* susceptible (Su, Ref), FA19 (Ref, square) and resistant (Re, Pathog, cross) strains. MIC were performed 3 times independently, using 2-fold serial dilutions of the antibiotics. When necessary, ranges (as color-coded bars) of MIC are displayed for specific data points that are plotted as a midpoint of the range.

**Figure 4:**
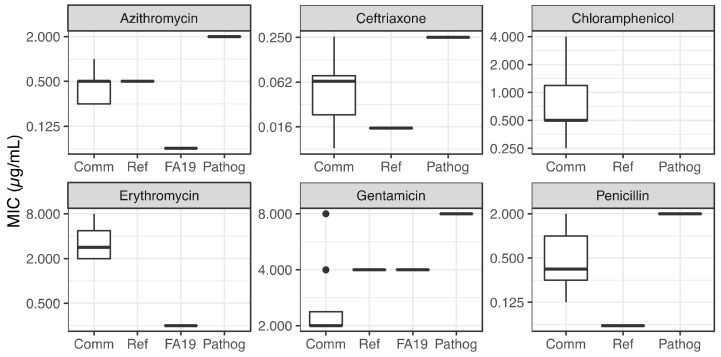
One-way ANOVA analysis of MIC (µg/mL) for each antibiotic per group (commensal, reference, *N. gonorrhoeae* FA19, pathogenic resistant). Scales are provided as log2.

Next, we performed minimal inhibitory concentration (MIC) assays using a 2-fold serial dilution on GCB plates of azithromycin, ceftriaxone, penicillin, erythromycin, chloramphenicol, and gentamicin (see materials and methods for concentration ranges). We compared our results to susceptible and resistant strains of *N. gonorrhoeae* with values reported in the literature. Figure 3 shows differences in MIC to these 6 antibiotics for the commensal *Neisseria* species tested in the lab. Figure 4, Table 3 and Table 4 report the results of the One-way ANOVA tests comparing the group of commensals to the reference. Similarly to our DDA analysis, commensal *Neisseria* species displayed a wide range of MIC levels. *N. lactamica* appeared more susceptible compared to the other commensal *Neisseria* species (Figure 3 and Table S2). Indeed, *N. cinerea*, *N. mucosa*, and *N. elongata* showed increased ceftriaxone MIC values (0.128 µg/mL, 0.064 µg/mL, 0.064 µg/mL, respectively), reflecting increased resistance, compared to *N. lactamica* (0.008 µg/mL) or a susceptible *N. gonorrhoeae* strain (≤0.015 µg/mL). *N. cinerea* displayed ceftriaxone MIC values 16 times higher than *N. lactamica*; *N. mucosa* and *N. elongata* displayed ceftriaxone MIC values 8 times higher than *N. lactamica*. However, these values of MIC for commensal *Neisseria* were still below the MIC breakpoint for ceftriaxone described by Kirkcaldy et al. [25]. The MIC values for azithromycin in commensal *Neisseria* were lower (0.25–0.5 µg/mL) than the MIC breakpoint for resistant *N. gonorrhoeae* (≥2 µg/mL) described by Kirkcaldy et al. [25]; hence, commensals appeared sensitive to azithromycin, detected by MIC assays, still more resistant than *N. gonorrhoeae* FA19 [26]. All commensals were overall more resistant to penicillin than the susceptible strain of *N. gonorrhoeae*. *N. elongata* and *N. mucosa* appeared more resistant than *N. cinerea* and *N. lactamica* towards chloramphenicol and erythromycin; we did not find MIC values to these antibiotics for *N. gonorrhoeae* susceptible and resistant strains, we only found the erythromycin MIC value for *N. gonorrhoeae* FA19 [26]. These observations reinforce the need to analyze genomic sequences of commensal *Neisseria* to identify possible antimicrobial resistance genes and mutations.

We performed a One-way ANOVA test (alpha level 1 × 10^−4^) on groups, for MIC data. Using MIC, we show that commensals tend to be more resistant to 4 antibiotics (except chloramphenicol and gentamicin) of the 6 tested compared to a reference *N. gonorrhoeae* susceptible strain. However, while erythromycin MIC showed statistically significant differences, the difference was not statistically significant for azithromycin, penicillin and ceftriaxone. One explanation is the great phenotypic variability within the group of commensals. The MIC results show that all commensals were more resistant to erythromycin than *N. gonorrhoeae* FA19 [26] (*p*-value < 1 × 10^−5^). For other antibiotics, MIC results do not display statistically significant differences between commensals and the reference, but show a trend where the commensals are more resistant than the reference (Figure 4, Table 3 and Table 4), in agreement with DDA results. In regard to chloramphenicol, we could only do a within-commensal group comparison. We observed that *N. mucosa* is more resistant to chloramphenicol than the other commensals (*p*-value < 1 × 10^−5^).

### 2.3. Mutations in Penicillin-Binding Protein 2 (PBP2) and other Protein Sequences Can Partially Explain Resistance in Commensal Species

In order to explore the potential mechanistic basis for variation in resistance among *Neisseria* species, we compared protein sequence variation amongst commensal and pathogenic strains, focusing on previously identified genes associated with antibiotic resistance. One of these genes, *penA*, encodes the penicillin-binding-protein-2 (PBP2), known to modify penicillin and other beta-lactam drugs [27]. In *N. gonorrhoeae*, known mutations in *penA* lead to increased resistance [28]. *N. gonorrhoeae* resistant strains often contain nucleotide point mutations leading to amino acid changes, and/or contain a mosaic sequence of *penA*, which is a recombination of *penA* genes from *N. perflava* and *N. cinerea* [29,30,31,32], both commensal species regularly carried in the human oro- and nasopharynx, where other commensal *Neisseria* also reside, and where *N. gonorrhoeae* may be found in cases of pharyngeal gonococcal infections.

**Figure 5:**
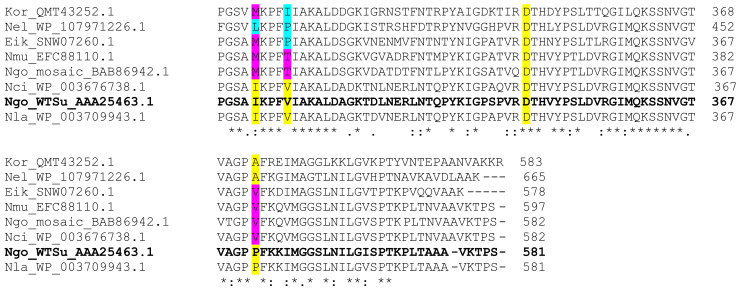
Multiple sequence alignment using ClustalOmega of portions of the PBP2 sequences from 2 *N. gonorrhoeae* strains, 4 commensal *Neisseria* species, and 2 non-*Neisseria* species from the Neisseriaceae family. The sequence for *N. gonorrhoeae* LM306 susceptible strain is bolded. Amino acid positions identified as relevant for antibiotic resistance (highlighted in yellow) that are mutated in more than one commensal are highlighted in magenta (known mutations), or cyan (undescribed mutations). Kor, *Kingella oralis*, Nel, *N. elongata*; Eik, *Eikenella corrodens*; Nmu, *N. mucosa*; Ngo, *N. gonorrhoeae*; Nci, *N. cinerea;* Nla, *N. lactamica*; WTSu, wild-type susceptible. * (asterisk) indicates identity for all sequences at that position; : (colon) indicates conservation by strong similarity among sequences at that position; . (period) indicates conservation by weak similarity among sequences at that position [34].

The *penA* sequences of commensal *Neisseria* species reveal genetic polymorphism. We aligned amino acid sequences of PBP2 from the different commensal *Neisseria*, *N. gonorrhoeae* susceptible strain LM306 (wild-type sequence, Ngo_WTSu) and resistant strain NG-3 (mosaic sequence, Ngo_mosaic), and 2 outgroup sequences from *Eikenella corrodens* (Eik) and *Kingella oralis* (Kor) (from the family Neisseriaceae), using BLASTp [33] and Clustal Omega [34] algorithms. We observed that mutations in PBP2 known to increase antibiotic resistance in *N. gonorrhoeae*, were present in *N. mucosa* (Nmu), *N. elongata* (Nel), and *N. cinerea* (Nci) (Figure 5). On the other hand, *N. lactamica* (Nla) conserved the amino acids present in the susceptible *N. gonorrhoeae* strain LM306 (highlighted in yellow). Other described mutations were not present in the commensal *Neisseria* species (hence, are not shown in Figure 5), namely the insertion of an aspartate after position 345 of the wild-type susceptible sequence [30,35], and the mutations A501V/P [36,37], and G545S [38].

The differences observed among PBP2 sequences of commensal *Neisseria* species may partially explain the higher resistance levels observed in these species for beta-lactam antibiotics, penicillin, ampicillin, and ceftriaxone. Other genes are likely involved for beta-lactam and other antibiotic resistance. However, the causal relationship between genes, mutations, and antimicrobial resistance are less clear than for *penA*. Supplementary Table S3 summarizes mutations observed in AR genes of commensal *Neisseria*, *Eikenella corrodens* and *Kingella oralis*. We used *rplD* and *rplV* as examples for macrolide resistance, *mtrR*, *ponA*, and *porB* for cephalosporin resistance, *rpsJ* and plasmid-carried *tetM* for tetracycline resistance, plasmid-carried chloramphenicol acetyltransferase (*cat*) for chloramphenicol resistance, and plasmid-carried *str* for streptomycin resistance. We were not able to identify genes or specific mutations described for gentamicin resistance. We observed that the majority (11/11) of the commensal sequences analyzed through BLASTp and the MSA viewer (NCBI) contained the mutations L6Q, D94N, R99Q, V17A, V45Q, T72V, S101C in *rplV*. Mutations K74S, D91N, V120A, V121I, K123A/S/D/E, T173Q/H, A190K/R in *rplD* were observed in 26/26 commensal sequences, associated with macrolide resistance [39]. Kanamycin and streptomycin resistance genes are carried by transposons that were not observed in the commensal sequences studied. The genes *tetM* and *cat* are known to be carried by plasmids, which have not been described for the commensal *Neisseria* strains studied. Overall, these observations are not enough to explain the vast intrinsic antimicrobial resistance by commensal *Neisseria* species described in this study. Hence, further analyses of known antimicrobial resistance genes and genomic data followed by mutagenesis and phenotypic analysis are necessary to understand and explain antibiotic resistance for non-beta-lactam antibiotics, such as azithromycin, chloramphenicol, and erythromycin.

## 3. Discussion

In this study, we characterized the antimicrobial resistance profiles of 4 commensal *Neisseria* species, closely related to pathogenic *Neisseria*, and clinically relevant given their natural niche, the human oral and nasal pharynx (ONP) [40]. We compared the resistance levels of *N. lactamica*, *N. cinerea*, *N. mucosa*, and *N. elongata* to 4 *N. gonorrhoeae* strains (FA19, MS11, F89, H041). We observed that *N. cinerea*, *N. mucosa*, and *N. elongata* generally displayed higher resistance levels than *N. gonorrhoeae* FA19 or *N. lactamica*. Given the high antimicrobial resistance (AR) profiles observed for the commensals, it is possible that commensals express AR genes, mutations, and/or mechanisms not yet identified in *N. gonorrhoeae*. Hence, commensals are likely antimicrobial resistance gene reservoirs for *N. gonorrhoeae*, particularly for azithromycin and erythromycin.

Among the ten antibiotics tested, ceftriaxone and azithromycin are particularly relevant as they are the current line of treatment against *N. gonorrhoeae*. Indeed, *N. gonorrhoeae* is treated with a dual therapy of oral azithromycin (1 g, single dose) and injectable intramuscular ceftriaxone (0.25 g, single dose) (or oral cefixime) [41], to which certain gonococcal strains display resistance, such as *N. gonorrhoeae* F89 and H041 from France and Japan, respectively [10,36]. While antibiotic resistance to this dual treatment is increasing, the rates of resistance are still below the 5% threshold needed to recommend new guidelines by the CDC. However, if this treatment fails, clinicians require a time- and resource-consuming antibiogram to assess the antibiotic susceptibility panel of the infectious strain. Gentamicin was the only antibiotic for which all strains displayed a similar level of susceptibility and were categorized as susceptible according to EUCAST and Bala et al. [24]. Gentamicin could be a possible drug of choice to treat extreme drug resistant *N. gonorrhoeae*, as all strains tested in our study show ZoI diameters larger than 16 mm, breakpoint for susceptibility to gentamicin [24]. Additionally, new data associated with gentamicin shows that *N. gonorrhoeae* WHO reference strains present an MIC of 4 µg/mL (dispersion 2–8 µg/mL) [24], which is also consistent with our MIC results for commensal *Neisseria*. However, gentamicin is an aminoglycoside used to treat Gram-negative infections causing bone, urinary tract and respiratory infections, endocarditis, meningitis, and pelvic inflammatory disease. Often, these infections have limited treatment alternatives. Knowing how easily *N. gonorrhoeae* acquires resistance whether through mutation or natural competence, it is absolutely indispensable that scientists and clinicians reflect on and model the outcome of prescribing gentamicin as a future drug of choice for *N. gonorrhoeae*. In addition, a recent report demonstrate that gentamicin as monotherapy is not a good alternative to treat pharyngeal gonorrhea [42].

In order to explore a potential genetic mechanism for variation in antibiotic resistance profiles, we analyzed protein sequences of PBP2, encoded by the *penA* gene, and other AR genes, in the four commensal strains along with the focal *N. gonorrhoeae* strain. We observed that mutations known to increase antibiotic resistance to beta-lactams were found in *N. cinerea*, *N. mucosa*, and *N. elongata*. However, these *penA* mutations were not found in *N. lactamica* which conserves the amino acids present in susceptible strains of *N. gonorrhoeae* strain LM306. These differences may partially explain the reduced susceptibility to beta-lactams observed in the commensals studied. In addition, the analysis of other possible AR gene mutations were also observed in commensal *Neisseria* species. This suggests that genomic analyses of commensal *Neisseria* species for antimicrobial resistance mechanisms could reveal known and new candidate genes, and mutations involved in antimicrobial resistance, in particular to azithromycin and ceftriaxone, first-line treatment antibiotics to treat *N. gonorrhoeae* infections. Further work is needed to explore the potential influence of plasmid-mediated penicillin resistance in commensal *Neisseria* species, which is highly prevalent in *N. gonorrhoeae* strains.

As with PBP2, several other genes are involved in antimicrobial resistance, such as *mtrR*, *penA* (PBP2), *penC*, *ponA* (PBP1), *tetM*, *pilTQ*, *folP*, *mtrC*, *23S rRNA*, *rpsJ*, *16S rRNA*, *gyrB*, *gyrA*, *parC*, *prld*, *porB* (PIB). Genetic analysis of these sequences for commensal *Neisseria* both at the nucleic acid and protein levels could help inform the antimicrobial resistance profile of the pathogen. Similarly, the analysis of gene expression of these genes in commensals under sub-MIC conditions could offer additional insight in the evolution of AR mechanisms and their regulation. Antibiotic resistance in commensals has been observed in several genera, often through transformation with plasmids carrying antibiotic resistance genes [43,44]. Antibiotic resistance is widely spread in *N. gonorrhoeae*, and researchers have focused their studies on genomic analyses of AR genes in pathogenic *Neisseria* [11]. However, given the potential for transfer of AR genes between *Neisseria* species [45,46,47,48,49], our results and those of Fiore et al. [39] emphasize the need to further investigate AR gene pools in commensal *Neisseria* species. Fiore et al. [39] initiated this work by performing a genomic and phenotypic study of the CDC AR panel of *Neisseria* species, using 6 antibiotics (penicillin, cefixime, ceftriaxone, tetracycline, azithromycin, and ciprofloxacin). Our study further complements that work by analyzing the antibiotic resistance profile of 8 *Neisseria* strains to 10 antibiotics. Together, these studies provide a gateway to understanding commensal bacteria mechanisms of resistance and help identify putative genes involved in the expression and regulation of these mechanisms.

Moving forward, given observations that antibiotic resistance levels were higher in *N. cinerea*, *N. mucosa*, and *N. elongata*, it would be critical to further compare genomic sequences of these species, to identify candidate genes involved in resistance mechanisms not yet observed in *N. gonorrhoeae*, and to continue the analysis of phenotypic antibiotic resistance level characterization of other commensal *Neisseria* species.

## 4. Materials and Methods

### 4.1. Bacterial Strains

Commensal and pathogenic *Neisseria* species were kindly provided by W.M. Shafer (Emory University School of Medicine, Atlanta, GA) and E. Aho (Concordia College, Moorhead, MN, USA). *N. lactamica* strain NRL 36,016 [50], *N. cinerea* strain ATCC 14685, *N. mucosa* strain NRL 9297 [50], and *N. elongata* strain ATCC 25295, *N. gonorrhoeae* FA19 [22], *N. gonorrhoeae* MS11 [51], *N. gonorrhoeae* F89 [36], *N. gonorrhoeae* H041 [10] were stored at −80 °C in GCB broth containing 30% glycerol. Bacteria were plated on GCB agar with supplements I and II [15], and incubated overnight at 37 °C, in a 5% CO_2_ atmosphere. When cultured in liquid broth, GCB liquid media was supplemented with supplements I and II, and 0.043% (*w*/*v*) of NaHCO_3_.

### 4.2. Disk Diffusion Assays

*Neisseria* strains plated on GCB agar plates, overnight, at 37 °C in 5% CO_2_ atmosphere, were harvested with a sterile loop and suspended in supplemented GCB broth at OD_600nm_ 0.2 UA. Bacteria were spread on the plate using CLSI guidelines [52]. Briefly, a sterile cotton swab was dipped in the suspension and spread in one direction on the plate. The procedure (dip and spread) was repeated two additional times, every time rotating the plate by 120 degrees, to obtain a homogeneous lawn of bacterial growth throughout the plate. Plates were then allowed to dry for 10 min, and antibiotic disks were applied. To prevent overlay of antibiotics or zones of inhibitions (ZoI), we applied 3 antibiotics per plate. Zones of inhibition were measured using AntibiogramJ [53] and ImageJ [54]. Averages and standard deviation (SD) values were obtained from 3 independent experiments, each containing 3 biological replicates. All strains (4 commensal strains and 4 *N. gonorrhoeae* strains (FA19, MS11, F89 and H041), were tested against a panel of 10 antibiotics (Penicillin 10 UI, Kanamycin 30 µg, Streptomycin 10 µg, Azithromycin 15 µg, Ceftriaxone 30 µg, Erythromycin 15 µg, Tetracycline 30 µg, Ampicillin 10 µg, Chloramphenicol 30 µg, Gentamicin 10 µg), on pre-loaded disks (6 mm diameter) purchased from Hardy Diagnostics (Santa Maria, CA, USA), stored at −20 °C when not in use.

### 4.3. Minimal Inhibitory Concentrations

The minimal inhibitory concentration (MIC) was determined as the concentration of antimicrobial inhibiting 99.99% (or a 4-log_10_ decrease) of bacterial growth. We used the plate dilution technique to quantify inhibition, following CLSI guidelines [55]. Briefly, three biological replicates of each bacterial species grown overnight on supplemented GCB plates at 37 °C, in 5% CO_2_, were harvested with sterile plastic loops, and suspended in supplemented GCB broth, at OD_600nm_ 0.2 UA. Five µL of each suspension were plated in triplicates (technical replicates), on supplemented GCB agar plates containing a range of antibiotics, in 2-fold serial dilutions. Plates contained 0.006 µg/mL to 2 µg/mL of Penicillin G, or 4 ng/mL to 512 ng/mL of Ceftriaxone, or 0.031 µg/mL to 4 µg/mL of Azithromycin, or 2 µg/mL to 256 µg/mL of Erythromycin, or 0.125 µg/mL to 16 µg/mL of Chloramphenicol, or 1 µg/mL to 64 µg/mL of Gentamicin. Plates were stored at 4 °C and used at most 5 days after plating. Three independent experiments were performed. The concentrations of antibiotics on plates were considered the minimal inhibitory concentrations where less than 4 colonies were observed per spot, as it suggests 99.99% growth inhibition.

### 4.4. Penicillin-Binding Protein 2 (PBP2) Sequence Alignment

Sequences from penicillin-binding protein 2 (PBP2) of several *Neisseria* and non-*Neisseria* Neisseriaceae sequences were obtained from NCBI. Amino acid sequences were aligned using ClustalOmega and BLASTp platforms, using the default parameters.

Nucleotide sequence accession numbers used were *N. gonorrhoeae* LM306 (AAA25463), *N. gonorrhoeae* NG-3 (BAB86942), *N. lactamica* (WP_003709943), *N. cinerea* (WP_003676738), *N. mucosa* (EFC88110), *N. elongata* (WP_107971226), *Kingella oralis* (QMT43252), *Eikenella corrodens* (SNW07260).

### 4.5. Data Analysis

Data were analyzed using R and RStudio [56], and plots were made using ggplot2 [57]. All experiments were performed at least 3 times independently. Each experiment contained 3 biological replicates of each species. Means and standard error of the means (SEM) were reported where applicable on the charts and tables. One-way ANOVA tests were used for DDA and MIC tests as described in Section 2. We used a conservative alpha threshold of 1 × 10^−4^ given the number of pairwise comparisons performed per antibiotic.

## 5. Conclusions

Here, we explored the antimicrobial resistance profiles of commensal *Neisseria* species. We demonstrated that several commensal *Neisseria* species express high levels of resistance to antimicrobial compounds, in particular azithromycin and ceftriaxone, which form the current dual-therapy treatment against *N. gonorrhoeae*. We propose that commensal *Neisseria* species found in the human oral and naso-pharynx can be reservoirs of antimicrobial resistance genes for pharyngeal *N. gonorrhoeae*, which could increase the spread of antimicrobial resistance among already hard-to-treat *N. gonorrhoeae*. In particular, we observed that *N. cinerea* and *N. elongata* were particularly resistant to several antimicrobials used to treat *N. gonorrhoeae*, to levels higher than known *N. gonorrhoeae* resistant strains such as MS11, F89, and H041. Further genomics and transcriptomics analysis are needed to pinpoint the genetic and gene regulation bases for these antimicrobial resistance profiles.

## Figures and Tables

**Table 1:** One-way ANOVA tests for zone of inhibition (ZoI) diameters (mm) of each antibiotic comparing 3 groups of bacteria (commensal, reference, pathogenic).

Antibiotic		Df	Sum of Sq	Mean Sq	F-Value	Pr (>F)
Ampicillin	group	2	1362	680.8	52.66	<1 × 10^−4^
	Residuals	105	1358	12.9		S
Azithromycin	group	2	4040	2020.2	273.6	<1 × 10^−4^
	Residuals	102	753	7.4		S
Ceftriaxone	group	2	2614	1306.9	126.2	<1 × 10^−4^
	Residuals	105	1087	10.4		S
Chloramphenicol	group	2	1708.4	854.2	98.43	<1 × 10^−4^
	Residuals	105	911.2	8.7		S
Erythromycin	group	2	2829	1414.5	277.6	<1 × 10^−4^
	Residuals	105	535	5.1		S
Gentamicin	group	2	159.2	79.59	44.21	<1 × 10^−4^
	Residuals	357	642.8	1.8		S
Kanamycin	group	2	653.1	326.5	36.57	<1 × 10^−4^
	Residuals	105	937.5	8.9		S
Penicillin	group	2	2851.8	1425.9	162.2	<1 × 10^−4^
	Residuals	105	922.9	8.8		S
Streptomycin	group	2	147.7	73.83	7.482	9.17 × 10^−4^
	Residuals	105	1036.1	9.87		NS
Tetracycline	group	2	818.1	409	83.72	<1 × 10^−4^
	Residuals	105	513	4.9		S

**Table 2:** One-way ANOVA multiple pairwise comparisons displaying the difference and the adjusted *p*-value of ZoI diameter means for 3 groups (commensal, reference, pathogenic) for each antibiotic. For this analysis, we selected a conservative alpha threshold of <1 × 10^−4^ given that we are comparing 10 different antibiotics.

**Antibiotic**	**Ampicillin**		**Azithromycin**		**Ceftriaxone**		**Chloramphenicol**		**Erythromycin**	
	**diff**	***p* adj**	**diff**	***p* adj**	**diff**	***p* adj**	**diff**	***p* adj**	**diff**	***p* adj**
Reference to commensal	12.807	<1 × 10^−4^	15.295	<1 × 10^−4^	14.866	<1 × 10^−4^	13.645	<1 × 10^−4^	15.51	<1 × 10^−4^
Pathogenic to commensal	−0.062	0.9962	12.631	<1 × 10^−4^	−3.268	<1 × 10^−4^	−1.014	0.23	9.566	<1 × 10^−4^
Pathogenic to reference	−12.869	<1 × 10^−4^	−2.665	0.0195	−18.134	<1 × 10^−4^	−14.659	<1 × 10^−4^	−5.943	<1 × 10^−4^
**Antibiotic**	**Gentamicin**		**Kanamycin**		**Penicillin**		**Streptomycin**		**Tetracycline**	
	**diff**	***p* adj**	**diff**	***p* adj**	**diff**	***p* adj**	**diff**	***p* adj**	**diff**	***p* adj**
Reference to commensal	0.733	0.0033	7.108	<1 × 10^−4^	15.206	<1 × 10^−4^	0.389	0.941	10.592	<1 × 10^−4^
Pathogenic to commensal	−1.141	<1 × 10^−4^	4.741	<1 × 10^−4^	−3.683	<1 × 10^−4^	−2.285	0.0021	2.743	<1 × 10^−4^
Pathogenic to reference	−1.874	<1 × 10^−4^	−2.367	0.0719	−18.889	<1 × 10^−4^	−2.674	0.0486	−7.85	<1 × 10^−4^

**Table 3:** Results of multiple pairwise comparisons of MIC means for 3 groups (commensal, reference, pathogenic) for each antibiotic.

Antibiotic		Df	Sum of Sq	Mean Sq	F-Value	Pr (>F)	Sign lev *
Azithromycin	group	3	12.61	4.202	7.912	1.61 × 10^−4^	NS
	Residuals	59	31.33	0.531			
Ceftriaxone	group	2	8.61	4.303	1.727	0.187	NS
	Residuals	59	147	2.492			
Erythromycin	group	1	13.68	13.682	19.35	9.75 × 10^−5^	S
	Residuals	35	24.75	0.707			
Gentamicin	group	3	39.59	13.196	44.52	<1 × 10^−4^	S
	Residuals	70	20.75	0.296			
Penicillin	group	2	13.16	6.582	3.619	0.043	NS
	Residuals	23	41.83	1.819			

* S, significance; NS, non-significance.

**Table 4:** Differences and adjusted *p*-values of the One-way ANOVA pairwise comparison between the reference susceptible and the commensal group.

Antibiotic	Reference-Commensal		FA19—Commensal	
	diff	*p* adj	diff	*p* adj
Penicillin	2.083	0.303		
Azithromycin	0.333	0.969	−2.655	3.41 × 10^−3^
Ceftriaxone	−1.593	0.579		
Erythromycin	NA	NA	−3.75	9.71 × 10^−5^
Gentamicin	0.583	0.717		
Chloramphenicol	NA	NA	NA	NA

NA, not available.

## Data Availability

Data and scripts can be accessed at https://doi.org/10.6084/m9.figshare.14511609.v1.

## Supplementary Materials

The following are available online at https://www.mdpi.com/article/10.3390/antibiotics10050538/s1, Table S1: Antibiotic Disk Diffusion Assay (DDA) results for 4 commensal Neisseria species and 4 N. gonorrhoeae strains, Table S2: Commensal Neisseria minimal inhibitory concentrations (MIC, µg/mL) to antibiotics (penicillin, azithromycin, ceftriaxone, erythromycin, chloramphenicol, gentamicin), Table S3: Known mutations confirmed or likely involved in antimicrobial resistance, with observed cognate mutations in commensal *Neisseria*.
