# Supplementary material for: Antimicrobial Resistance Profiles of Human Commensal Neisseria Species
Source: Antibiotics (Basel). 2021 May 6;10(5):538. doi: 10.3390/antibiotics10050538 (PMC8148603; doi:10.3390/antibiotics10050538)
Supplement: Supplementary file 1 [file antibiotics-10-00538-s001.zip › antibiotics-1170469-supplementary.pdf]

# Supplementary Materials:

**Table S1:** Antibiotic Disk Diffusion Assay (DDA) results for 4 commensal *Neisseria* species and 4 *N. gonorrhoeae* strains. Average (± SEM) diameter (mm) of the zones of inhibition from 3 biological replicates, of 3 independent experiments, were measured using ImageJ [2].

| | *Neisseria* species | | | | | | | |
|---|---|---|---|---|---|---|---|---|
| ZoI (mm) | *Nla* | *Nci* | *Nmu* | *Nel* | *Ngo* FA19[a] | *Ngo* MS11[b] | *Ngo F89*[c] | *Ngo H041*[d] |
| Penicillin -10 UI | 32.5 (±0.3) | 29.8 (±0.6) | 34.0 (±0.3) | 29.7 (±0.3) | 46.7 (±1.0) | 26.9 (±0.8) | 31.6 (±0.3) | 24.4 (±0.3) |
| Ampicillin -10ug | 31.6 (±1.0) | 28.3 (±0.3) | 29.2 (±1.0) | 29.5 (±0.4) | 42.5 (±0.4) | 30.5 (±0.1) | 34.2 (±0.5) | 24.8 (±0.3) |
| Ceftriaxone -30ug | 41.2 (±0.8) | 30.1 (±1.1) | 37.4 (±0.2) | 32.4 (±0.5) | 50.2 (±0.6) | 37.2 (±0.7) | 31.5 (±0.4) | 30.8 (±0.3) |
| Azithromycin -15ug | 24.6 (±0.7) | 15.5 (±0.3) | 21.4 (±0.3) | 20.9 (±0.2) | 35.9 (±0.8) | 31.5 (±0.4) | 33.0 (±1.1) | 34.2 (±0.3) |
| Erythromycin -15ug | 21.4 (±0.4) | 14.9 (±0.2) | 15.9 (±0.4) | 18.0 (±1.0) | 33.1 (±0.7) | 26.0 (±0.5) | 26.2 (±1.0) | 28.4 (±0.4) |
| Chloramphenicol -30ug | 35.1 (±1.2) | 25.6 (±1.2) | 27.3 (±0.4) | 26.3 (±0.4) | 42.2 (±0.5) | 24.8 (±0.5) | 29.7 (±0.6) | 26.3 (±0.2) |
| Kanamycin -30ug | 30.7 (±0.6) | 21.5 (±0.1) | 31.4 (±0.2) | 23.1 (±0.3) | 33.8 (±0.5) | 27.8 (±0.6) | 31.9 (±0.6) | 32.2 (±0.3) |
| Streptomycin -10ug | 18.7 (±0.3) | 14.0 (±0.2) | 19.7 (±0.4) | 13.8 (±0.4) | 16.9 (±0.5) | 6.0 (±0.1) | 15.2 (±0.4) | 16.1 (±0.3) |
| Tetracycline -30ug | 32.8 (±0.5) | 25.6 (±0.4) | 24.9 (±0.4) | 25.3 (±0.2) | 37.8 (±0.6) | 28.0 (±0.4) | 30.8 (±0.4) | 29.7 (±0.3) |
| Gentamicin -10ug | 19.9 (±0.3) | 17.8 (±0.2) | 21.8 (±0.3) | 17.9 (±0.2) | 20.2 (±0.2) | 17.5 (±0.3) | 18.2 (±0.4) | 19.0 (±0.1) |

Zones of inhibition (ZoI) of and similar (<20% diameter reduction) to *N. gonorrhoeae* FA19 are highlighted in grey. ZoI highlighted in pink are > 33% smaller than those of *N. gonorrhoeae* FA19, and denote high resistance levels. Intermediate species display ZoI smaller than those of *N. gonorrhoeae* FA19 by 20% to 33% and are highlighted in yellow. The antimicrobial disk measures 6 mm in diameter, which is the minimum ZoI measurable. Species: Nla, *N. lactamica*; Nci, *N. cinerea*; Nmu, *N. mucosa*; Nel, *N. elongata*; Ngo, *N. gonorrhoeae*

[a] *N. gonorrhoeae* FA19 is considered susceptible to most antibiotics presented, used as reference [3].

[b] *N. gonorrhoeae* MS11 is considered highly resistant to streptomycin [4].

[c] *N. gonorrhoeae* F89 is considered highly resistant to cefixime and ceftriaxone [5].

[d] *N. gonorrhoeae* H041 is considered highly resistant to ceftriaxone [6].

**Table S2:** Commensal *Neisseria* minimal inhibitory concentrations (MIC, µg/mL) to antibiotics (penicillin, azithromycin, ceftriaxone, erythromycin, chloramphenicol, gentamicin).

| MIC (µg/mL) | Penicillin | Ceftriaxone | Azithromycin | Erythromycin | Chloram phenicol | Genta micin |
|---|---|---|---|---|---|---|
| *N. lactamica* | 0.25 | 0.008 | 0.25 | 2 | 0.5 | 2 |
| *N. cinerea* | 0.125-0.25 | 0.128 | 0.25-0.5 | 2 | 0.5 | 2 |
| *N. mucosa* | 2 | 0.064 | 0.5 | 8 | 4 | 2 |
| *N. elongata* | 0.5-1 | 0.064 | 0.5 | 4 | 1 | 8 |
| *N. gonorrhoeae* FA19 | | | 0.063[a] | 0.25[a] | | 4 |
| *N. gonorrhoeae Su*[b] | ≤ 0.06 | ≤ 0.015 | ≤ 0.5 | NA | NA | 4 |
| *N. gonorrhoeae Re*[b] | ≥ 2 | ≥ 0.25[c] | ≥ 2[c] | NA | NA | 8 |

MIC values 16-fold or higher compared to susceptible *N. gonorrhoeae*'s values are highlighted in pink, while values 4-8 fold higher are highlighted in yellow.

[a] N. gonorrhoeae FA19 values according to Zarantonelli *et al.* [7].

[b] *N. gonorrhoeae*'s MIC breakpoint (µg/mL) values according to CLSI [8].

[c] *N. gonorrhoeae*'s MIC values of wild-type distribution according to Kirkcaldy *et al.* [9].

**Table S3:** Known mutations confirmed or likely involved in antimicrobial resistance, with observed cognate mutations in commensal *Neisseria*

| Antibiotic class | Antibiotic | gene | Known Mutation[a] | Observed Cognate Mutation in commensal *Neisseria* | Proportion present | Reference |
|---|---|---|---|---|---|---|
| Macrolide | Azithromycin, Erythromycin | rplV | I4L | | 0 | [10] |
| Macrolide | Azithromycin, Erythromycin | rplV | L6Q | L6Q | 9/11 | [10] |
| Macrolide | Azithromycin, Erythromycin | rplV | K6Q | | 0 | [10] |
| Macrolide | Azithromycin, Erythromycin | rplV | T72A | T72V | 11/11 | [10] |
| Macrolide | Azithromycin, Erythromycin | rplV | S101T | S101C | 11/11 | [10] |
| Macrolide | Azithromycin, Erythromycin | rplV | I103L | | 0 | [10] |
| Macrolide | Azithromycin, Erythromycin | rplV | K83N | | 0 | [10] |
| Macrolide | Azithromycin, Erythromycin | rplV | D94H | D94N | 11/11 | [10] |
| Macrolide | Azithromycin, Erythromycin | rplV | K98N | R99Q | 11/11 | [10] |
| Macrolide | Azithromycin, Erythromycin | rplV | V17I | V17A | 11/11 | [10] |
| Macrolide | Azithromycin, Erythromycin | rplV | L46Q | V45Q | 11/11 | [10] |
| Macrolide | Azithromycin, Erythromycin | rplD | V52I | | 0 | [10] |
| Macrolide | Azithromycin, Erythromycin | rplD | V52L | | 0 | [10] |
| Macrolide | Azithromycin, Erythromycin | rplD | A37S | | 0 | [10] |
| Macrolide | Azithromycin, Erythromycin | rplD | K74T | K74S | 26/26 | [10] |
| Macrolide | Azithromycin, Erythromycin | rplD | D91E | D91N | 26/26 | [10] |
| Macrolide | Azithromycin, Erythromycin | rplD | V120I | V120A | 9/26 | [10] |
| Macrolide | Azithromycin, Erythromycin | rplD | V120I | V121I | 10/26 | [10] |
| Macrolide | Azithromycin, Erythromycin | rplD | K123S | K123A | 14/26 | [10] |
| Macrolide | Azithromycin, Erythromycin | rplD | K123S | K123S | 1/26 | [10] |
| Macrolide | Azithromycin, Erythromycin | rplD | K123S | K123D | 1/26 | [10] |
| Macrolide | Azithromycin, Erythromycin | rplD | K123S | K123E | 14/26 | [10] |

| | | | | | | |
|---|---|---|---|---|---|---|
| Macrolide | Azithromycin, Erythromycin | rplD | D154E | | 0 | [10] |
| Macrolide | Azithromycin, Erythromycin | rplD | T173N | T173Q | 16/26 | [10] |
| Macrolide | Azithromycin, Erythromycin | rplD | T173N | T173H | 9/26 | [10] |
| Macrolide | Azithromycin, Erythromycin | rplD | A190V | A190K | 13/26 | [10] |
| Macrolide | Azithromycin, Erythromycin | rplD | A190V | A190R | 8/27 | [10] |
| Macrolide | Azithromycin, Erythromycin | mtrR | A39T | | | [11] |
| Macrolide | Azithromycin, Erythromycin | mtrR | G45D | | | [11] |
| Beta-lactams/ Cephalosporin | Penicillin, Ampicillin, Ceftriaxone | penA | F504L | | | [11] |
| Beta-lactams/ Cephalosporin | Penicillin, Ampicillin, Ceftriaxone | penA | A510V | | | [11] |
| Beta-lactams/ Cephalosporin | Penicillin, Ampicillin, Ceftriaxone | penA | A516G | | | [11] |
| Beta-lactams/ Cephalosporin | Penicillin, Ampicillin, Ceftriaxone | penA | P551L | | | [11] |
| Beta-lactams/ Cephalosporin | Penicillin, Ampicillin, Ceftriaxone | penA | I312M | | | [11] |
| Beta-lactams/ Cephalosporin | Penicillin, Ampicillin, Ceftriaxone | penA | V316T | | | [11] |
| Beta-lactams/ Cephalosporin | Penicillin, Ampicillin, Ceftriaxone | ponA | L421P | | | [11] |
| Beta-lactams/ Cephalosporin | Penicillin, Ampicillin, Ceftriaxone | porB | G120K | | 0 | [11] |
| Tetracycline | Tetracycline | rpsJ | V57M | | 0 | |
| Tetracycline | Tetracycline | tetM | plasmid mediated | Not observed | 0 | |
| Chloramphenicol | Chloramphenicol | CAT | plasmid mediated | Not observed | 0 | |
| Aminoglycoside | Streptomycin, Spectinomycin, Kanamycin | str | | Not observed | 0 | |
| Aminoglycoside | Gentamicin | N/A | | Not observed | 0 | |

[a] Known mutations may have been identified in *N. gonorrhoeae* or in other model organisms such as *Escherichia coli*.

**References:**


1. Alonso, C.A.; Domínguez, C.; Heras, J.; Mata, E.; Pascual, V.; Torres, C.; Zarazaga, M. Antibiogramj: A tool for analysing images from disk diffusion tests. *Computer Methods and Programs in Biomedicine* **2017**, *143*, 159-169, doi:10.1016/j.cmpb.2017.03.010.
2. Schneider, C.A.; Rasband, W.S.; Eliceiri, K.W. NIH Image to ImageJ: 25 years of image analysis. *Nat Methods* **2012**, *9*, 671-675, doi:10.1038/nmeth.2089.
3. McKenna, W.R.; Mickelsen, P.A.; Sparling, P.F.; Dyer, D.W. Iron uptake from lactoferrin and transferrin by *Neisseria gonorrhoeae*. *Infect Immun* **1988**, *56*, 785-791, doi:10.1128/IAI.56.4.785-791.1988.
4. Swanson, J.; Barrera, O.; Sola, J.; Boslego, J. Expression of outer membrane protein II by gonococci in experimental gonorrhea. *J Exp Med* **1988**, *168*, 2121-2129, doi:10.1084/jem.168.6.2121.
5. Unemo, M.; Golparian, D.; Nicholas, R.; Ohnishi, M.; Gallay, A.; Sednaoui, P. High-level cefixime- and ceftriaxone-resistant *Neisseria gonorrhoeae* in France: novel *penA* mosaic allele in a successful international clone causes treatment failure. *Antimicrob Agents Chemother* **2012**, *56*, 1273-1280, doi:10.1128/AAC.05760-11.
6. Ohnishi, M.; Golparian, D.; Shimuta, K.; Saika, T.; Hoshina, S.; Iwasaku, K.; Nakayama, S.; Kitawaki, J.; Unemo, M. Is *Neisseria gonorrhoeae* initiating a future era of untreatable gonorrhea?: detailed characterization of the first strain with high-level resistance to ceftriaxone. *Antimicrob Agents Chemother* **2011**, *55*, 3538-3545, doi:10.1128/AAC.00325-11.
7. Zarantonelli, L.; Borthagaray, G.; Lee, E.H.; Veal, W.; Shafer, W.M. Decreased susceptibility to azithromycin and erythromycin mediated by a novel *mtrR* promoter mutation in *Neisseria gonorrhoeae*. *J Antimicrob Chemother* **2001**, *47*, 651-654.
8. Clinical and Laboratory Standards Institute. Performance Standards for Antimicrobial Susceptibility Testing; Twenty-Third Informational Supplement. **2013**.
9. Kirkcaldy, R.D.; Harvey, A.; Papp, J.R.; Del Rio, C.; Soge, O.O.; Holmes, K.K.; Hook, E.W., 3rd; Kubin, G.; Riedel, S.; Zenilman, J.; et al. *Neisseria gonorrhoeae* Antimicrobial Susceptibility Surveillance - The Gonococcal Isolate Surveillance Project, 27 Sites, United States, 2014. *MMWR Surveill Summ* **2016**, *65*, 1-19, doi:10.15585/mmwr.ss6507a1.
10. Fiore, M.A.; Raisman, J.C.; Wong, N.H.; Hudson, A.O.; Wadsworth, C.B. Exploration of the *Neisseria* Resistome Reveals Resistance Mechanisms in Commensals That May Be Acquired by *N. gonorrhoeae* through Horizontal Gene Transfer. *Antibiotics (Basel)* **2020**, *9*, doi:10.3390/antibiotics9100656.
11. Liao, M.; Gu, W.M.; Yang, Y.; Dillon, J.A. Analysis of mutations in multiple loci of *Neisseria gonorrhoeae* isolates reveals effects of PIB, PBP2 and MtrR on reduced susceptibility to ceftriaxone. *J Antimicrob Chemother* **2011**, *66*, 1016-1023, doi:10.1093/jac/dkr021.
